# Efficacy of a novel microneedle patch for rejuvenation of the nasolabial fold

**DOI:** 10.1111/srt.13199

**Published:** 2022-08-16

**Authors:** Ungsuthorn Pruettijarai, Jitlada Meephansan, Ornicha Prapapan, Purit Pureesrisak, Punyaphat Sirithanabadeekul, Kittipong Tantisantisom, Sattra Thongma, Yossawat Rayanasukha, Punyanuch Adulyaritthikul, Paisan Khanchaitit

**Affiliations:** ^1^ Division of Dermatology Chulabhorn International College of Medicine Thammasat University Pathum Thani Thailand; ^2^ Division of Dermatology Department of Medicine Rajavithi Hospital Ministry of Public Health Bangkok Thailand; ^3^ National Nanotechnology Center (NANOTEC) National Science and Technology Development Agency (NSTDA) Pathum Thani Thailand

**Keywords:** aging, hyaluronic acid, microneedle patch, rejuvenation, wrinkle

## Abstract

**Background:**

Skin rejuvenation plays a significant role in the esthetic medicine market. Microneedle patches have been developed for a wide range of applications based on the principles of transdermal drug delivery; however, clinical trials of microneedle patches for skin rejuvenation remain limited.

**Aims:**

This study was conducted to examine the efficacy of microneedle patches for improving nasolabial folds.

**Methods:**

A total of 23 Thai women completed this prospective clinical trial. The participants were treated according to a split‐face design, with application of microneedle patch plus 1.8% hyaluronic acid solution to the right nasolabial fold and microneedle patch alone to the left nasolabial fold. The treatments were applied to the nasolabial fold for 8 weeks. The test areas were measured before treatment and at 2, 4, 8, 12, and 16 weeks after the use of the test product.

**Results:**

Combination treatment using the microneedle patch plus hyaluronic acid solution and use of the microneedle patch alone both significantly improved the Merz esthetic scales for nasolabial folds. Measurement of the nasolabial fold showed an improvement in the two groups, with no significant differences between the groups. No adverse effects were reported during the study period.

**Conclusions:**

Application of a microneedle patch with 1.8% hyaluronic acid solution or a microneedle patch alone were both effective treatments for improving facial wrinkles in the nasolabial folds.

## 1 INTRODUCTION

Aging skin is a visible sign of physical aging.^1^ Wrinkles gradually appear as collagen, and elastic fibers decrease over time,^2^ and loss of subcutaneous fat and skin moisture accelerates the process.^3^ Thus, there is an increasing demand for skin rejuvenation with both surgical and nonsurgical procedures, the favorability of which depends upon minimal invasiveness and downtime. Currently, there are various kinds of noninvasive cosmetics available on the market; however, their limited efficacy is often attributed to the low skin penetration of active compounds due to the stratum corneum, which serves as an external barrier.^4^

The microneedle patch has been newly introduced for drug delivery through the skin^5^ and has been used to improve transdermal delivery of active agents, such as drugs and vaccines, in various medical fields.^6^ Since clinical trials of microneedle patches for skin rejuvenation remain limited, the purpose of this study was to measure the efficacy of a newly designed microneedle patch combined with hyaluronic acid (HA) for the improvement of the nasolabial fold.

## 2 MATERIALS AND METHODS

### 2.1 Study design

The study was designed as an investigator‐blinded, randomized controlled trial. The protocol was developed in accordance with the principles of good clinical practice and is within the applicable regulatory requirements. The Institutional Review Board of the Human Research Ethics Committee of Thammasat University (Medicine) approved all study protocols (number: MTU‐EC‐OO‐0‐078/64, November 25, 2021), and relevant supporting data. Written informed consent was obtained from all participants. No participants or dermatologists evaluated the clinical outcome of the treatment assignment, and blinding was maintained throughout the study.

### 2.2 Participants

Twenty‐three Thai women aged 30–60 years were recruited as volunteers. All volunteers had wrinkles in the nasolabial folds that were considered mild‐to‐moderate on the Merz esthetic scale. Exclusion criteria included any skin abnormalities such as infection and inflammation, history of adverse effects from polymethylmethacrylate (PMMA), use of energy‐based devices within 1 year, laser therapy within 6 months, mesotherapy within 1 month, filler injection in the nasolabial fold area within 1 year, current use of topical HA solution, and pregnancy or lactation. A microneedle patch plus HA was applied to the right side and a micronnedle patch alone was applied to the left side, referred to as groups 1 and 2, respectively. The treatments were each applied to the nasolabial fold for 8 weeks.

### 2.3 Microneedle patch and HA solution

**FIGURE 1:**
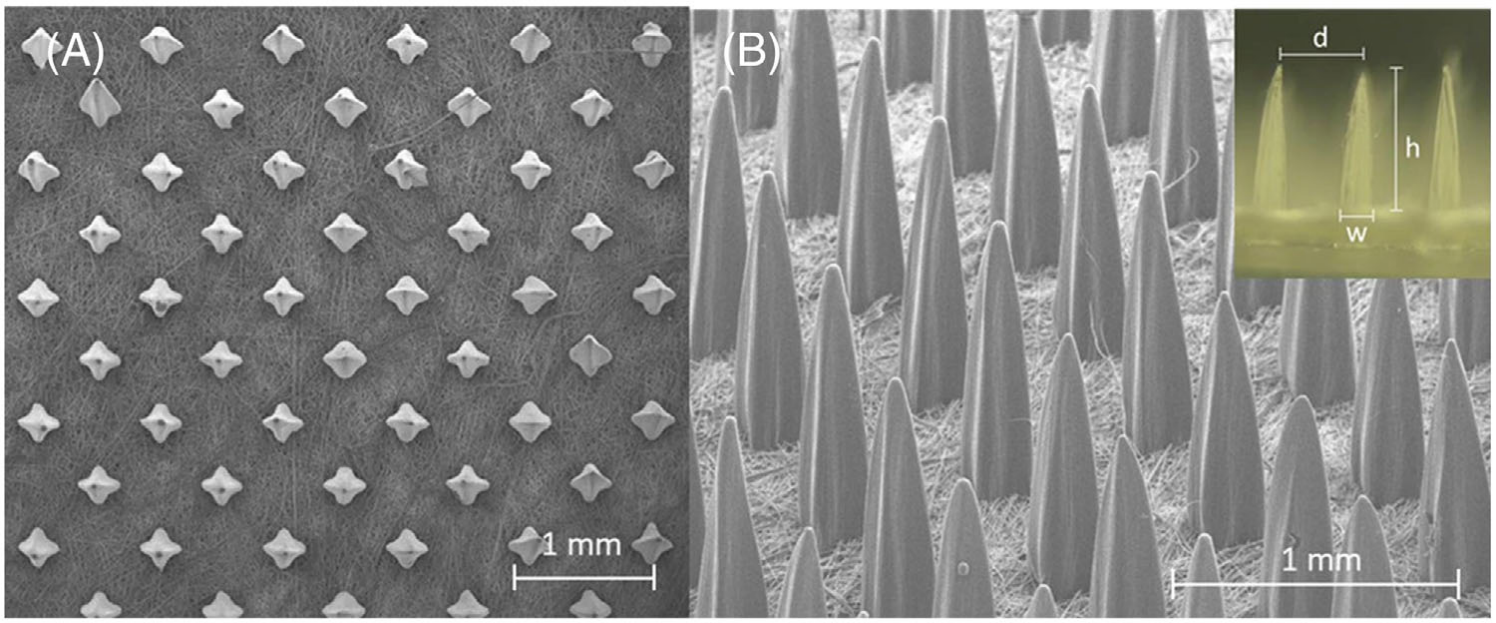
(A) Top view and (B) side view of the scanning electron microscope images of the four‐point star‐shaped microneedle array constructed on the fabric substrate. (Inset) An optical image showing the dimension parameters of the microneedle array

**FIGURE 2:**
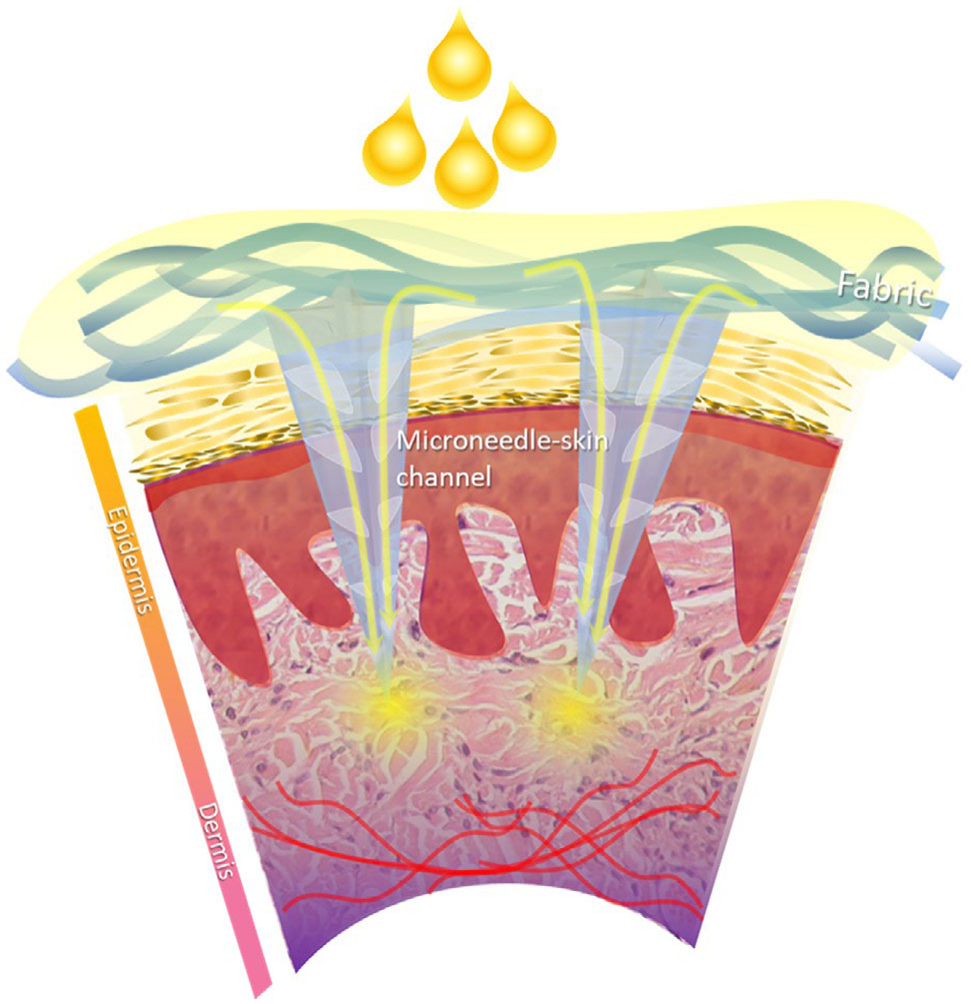
The active ingredient or solution delivery mechanism of the four‐point star‐shaped microneedle array fabricated on the fabric substrate

**FIGURE 3:**
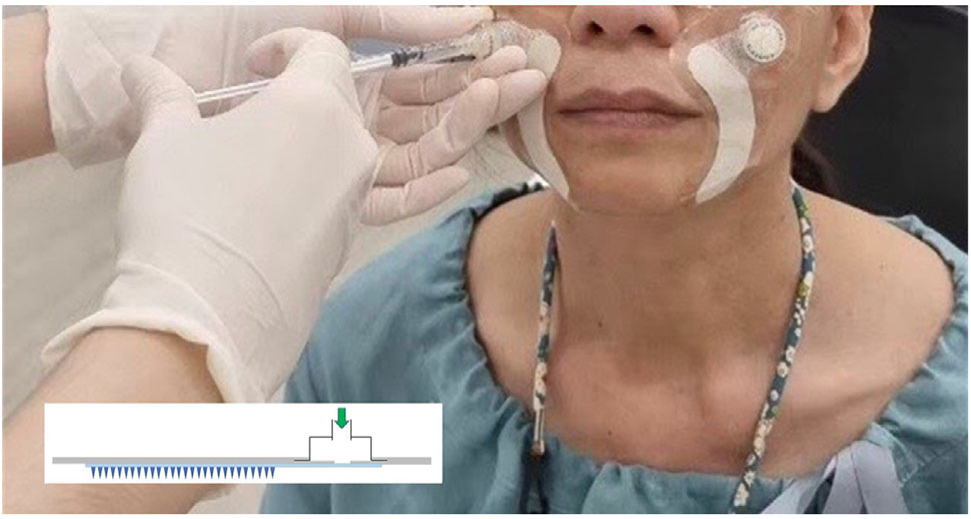
Image showing hyaluronic acid solution being pushed through the syringe connected with the microneedle patch. The insert shows the schematic structure of the microneedle patch used in this clinical study

The microneedle patches were prepared by the nanoneedle research team, responsive material and nanosensor research Group, National Nanotechnology Center, Thailand. The microneedle array was fabricated on the fabric substrate via the photo‐polymerization technique. Each microneedle was designed with a four‐point star shape, as shown in Figure 1A. This four‐point star structure is also shown from the side in Figure 1B, with a fin along each side of a microneedle. The average height (h) and base diameter (w) of the microneedles were 1100 and 280 μm, respectively. The distance between the tip of the microneedles (d) was 900 μm. The density of the microneedle array in this study was 265 needles/cm^2^. The shape of the microneedle patch was designed to cover the nasolabial fold area, and the total area of the microneedle array in the patch was 7.5 cm^2^. The active ingredient delivery mechanism of the microneedle array in the patch is presented in Figure 2. The fins of each four‐point star‐shaped microneedle formed a gap or channel between the microneedle grooves and tissue when it was punctured into skin. As seen in the schematic structure in Figure 3 (inset), a reservoir was attached to the patch, which connected to a syringe, allowing injection of the hyaluronic solution onto the fabric. The solution was thus absorbed and flowed through substrates along the channel formed by microneedles and surrounding tissue underneath the skin. The part of the microneedle patch that penetrated through the skin was sterile and was manufactured from polymers in the methacrylate group, such as PMMA, which is biocompatible and approved for application in clinical studies. Commercial 1.8% hyaluronic solution (Fusion F‐HA; Institute BCN esthetics, Spain) was provided to each participant in group 1.

### 2.4 Intervention

A microneedle patch with HA was applied to the nasolabial fold area of right side of the participant's face, as shown in Figure 3, while the microneedle patch alone was applied to the other side once every 2 weeks for a total of 8 weeks. All applications were performed after the face was washed, and no topical anesthetics were administered during the clinical trial. Participants were instructed to massage the patch on each side for 5 min and then remove it. Moisturizers were provided to each participant.

### 2.5 Quantitative evaluation of wrinkle improvement

The severity of wrinkles was assessed before patch application and again at 2, 4, 8, 12, and 16 weeks.

### 2.6 Subjective and objective assessments of wrinkle improvement

Two blinded dermatologists independently evaluated wrinkle grade according to the Merz scale by examining photographs taken at each visit; the assigned grades were recorded. Participants completed surveys with a 5‐grade scale for the improvement of facial wrinkles, comprising the following ratings: excellent (4), very improved (3), improved (2), no change (1), and worse (0). The efficacy of each intervention was evaluated based on the percentage of each response obtained in each group.

### 2.7 Safety and product preference

Patients were surveyed for development of any adverse effects, such as erythema, edema, itching, and irritation, at each visit. No adverse effects were reported.

### 2.8 Statistical analysis

All data are presented as mean ± SD. Statistical significance was set at *p* < 0.05. All statistical analyses were performed using Statistical Package for the Social Sciences software (Chicago, IL, USA). Generalized estimating equations were used for intragroup and intergroup comparisons.

## 3 RESULTS

### 3.1 Patient characteristics

The average age of the 23 participants who participated in this study was 50.78 ± 6.41 years. Nine people (39.13%) were in their 40s, 12 (52.17%) were in their 50s, and two (8.69%) were in their 60s. All participants were women.

### 3.2 Efficacy end points

**FIGURE 4:**
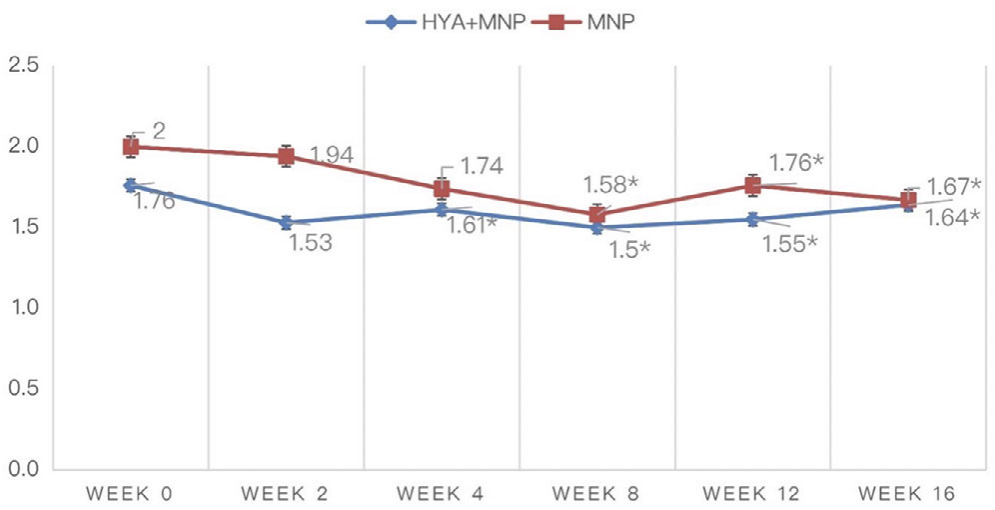
Nasolabial fold wrinkle grade assessed by independent blinded dermatologists (*p < 0.05 compared within groups)

**FIGURE 5:**
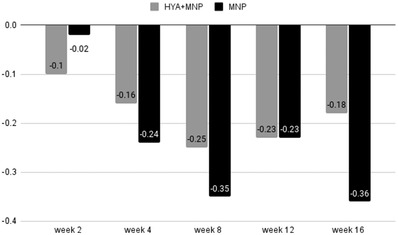
The change in nasolabial fold wrinkle grade assessed by independent blinded dermatologists between groups (*p < 0.05 compared within groups)

Before treatment, the results of the clinical assessment performed by two dermatologists using the Merz esthetics scale were 1.76 ± 0.62 in group 1 and 2 ± 0.74 in group 2 (0 = no folds, 1 = mild folds, 2 = moderate folds, 3 = severe folds, 4 = very severe folds). At weeks 2, 4, 8, 12, and 16, the average ratings were 1.53 ± 0.62, 1.61 ± 0.66, 1.5 ± 0.67, 1.55 ± 0.71, and 1.64 ± 0.7 in group 1, respectively. In comparison, those in group 2 were 1.94 ± 0.85, 1.74 ± 0.75, 1.58 ± 0.77, 1.76 ± 0.77, and 1.67 ± 0.77 at weeks 2, 4, 8, 12, and 16, respectively. The intraclass correlation coefficients of the two dermatologists were 0.608 and 0.624 for groups 1 and 2, respectively. The results from the Merz esthetics scale suggested that there was a statistically significant improvement in the scores from weeks 4 to 16 in both groups 1 and 2. There were no significant differences between the two groups. The results of the Merz esthetics scale are shown in Figure 4. The change in nasolabial fold wrinkle grade assessed by independent blinded dermatologists between groups compared with baseline is shown in Figure 5.

**FIGURE 6:**
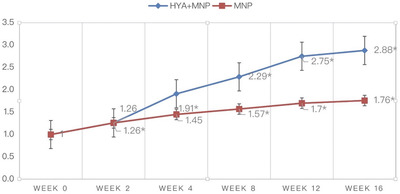
Nasolabial fold wrinkle improvement assessed by participants (*P<0.05 compared within group)

**FIGURE 7:**
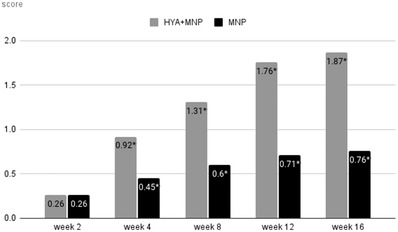
The change in nasolabial fold wrinkle improvement assessed by participants (*P < 0.05 via the generalized estimating equation)

**FIGURE 8:**
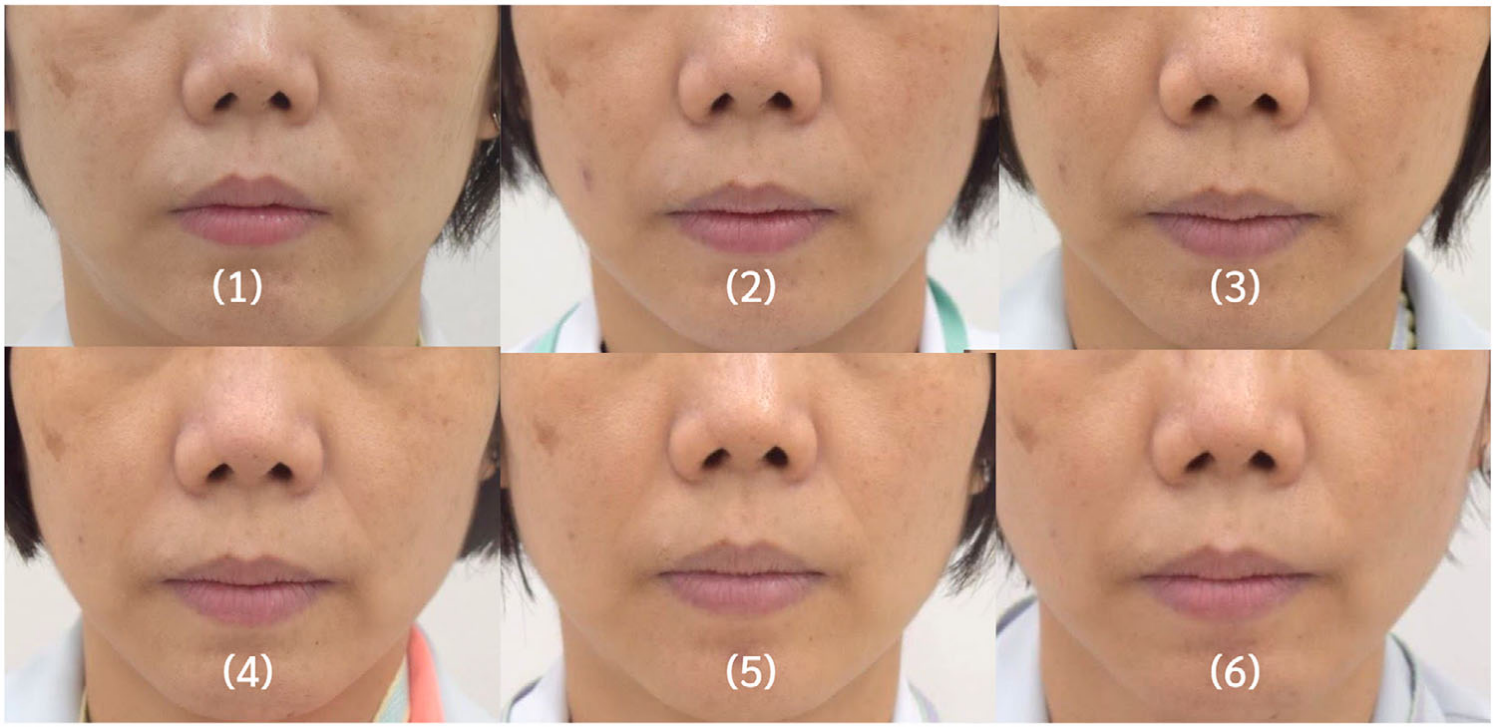
Images of nasolabial folds of representative cases in the microneedle patch plus hyaluronic acid (HA) solution (right side) and microneedle patch alone (left side) groups, at (1) baseline, and (2) 2, (3) 4, (4) 8, (5) 12, and (6) 16 weeks

**FIGURE 9:**
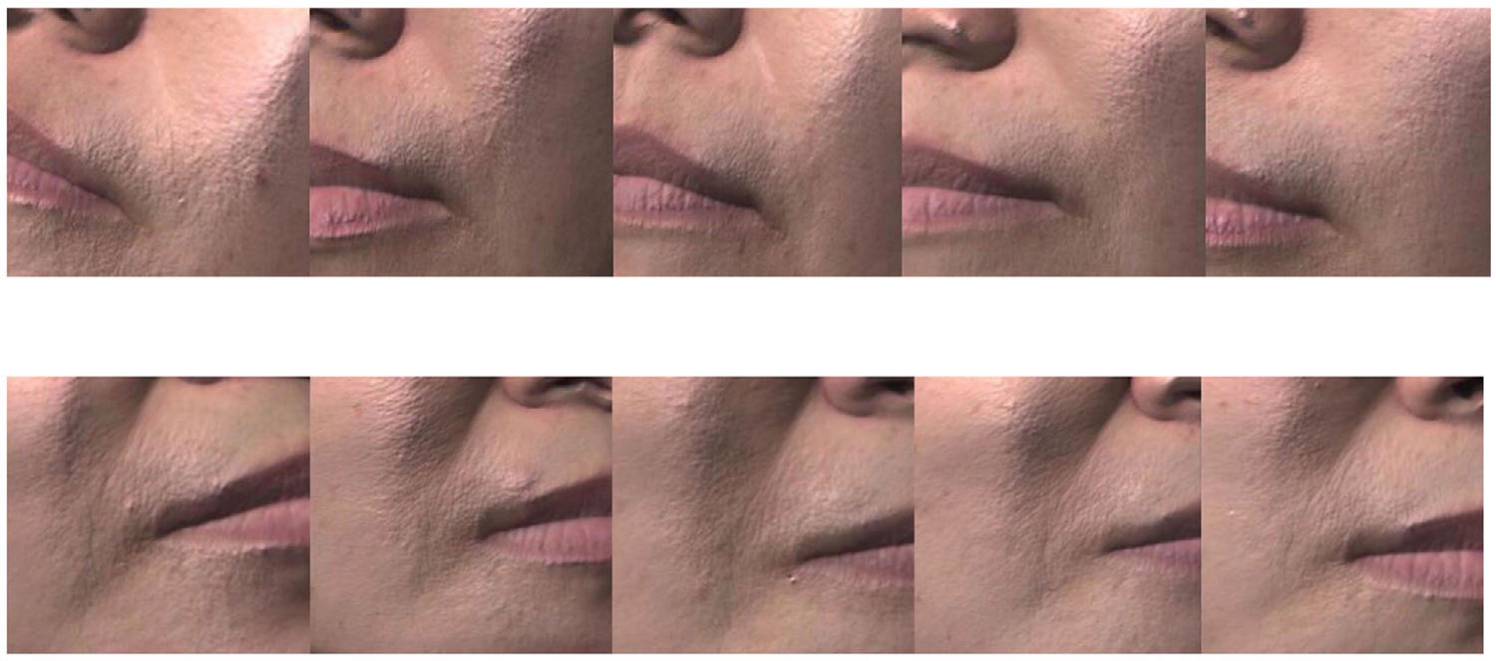
The change in nasolabial fold wrinkle improvement: Images of nasolabial folds of representative cases in the microneedle patch plus hyaluronic acid (HA) solution (right side) and microneedle patch alone (left side) at baseline, 4, 8, 12, and 16 weeks

In the participant‐reported surveys, the scores were 1 at baseline for both groups (0 = clinically worsened at any percentage, 1 = not clinically improved, 2 = clinically improved about 25%–50%, 3 = clinically improved about 50%–75%, 4 = clinically improved about 75%–100%). At weeks 2, 4, 8, 12, and 16, the mean scores were 1.26 ± 0.45, 1.91 ± 0.75, 2.29 ± 0.85, 2.75 ± 0.91, and 2.88 ± 0.86 in group 1, respectively. In comparison, those in group 2 were 1.26 ± 0.54, 1.45 ± 0.74, 1.57 ± 0.68, 1.7 ± 0.86, and 1.76 ± 0.9 at weeks 2, 4, 8, 12, and 16, respectively. The participants’ assessments suggested that there were statistically significant improvements from weeks 4 to 16 in group 1 and from weeks 2 to 16 in group 2. Moreover, there were significant differences between the two groups from weeks 4 to 16, indicating that group 1 had higher satisfaction with clinical outcomes. The details of the scores are shown in Figures 6. The change in nasolabial fold wrinkle improvement assessed by participants compared with baseline is shown in Figure 7. Clinical photographs are shown in Figures 8 and 9.

### 3.3 Safety end points

No adverse effects were reported by the participants during the application period of the test product, and no skin abnormalities were observed on physical examination by the dermatologist. In the survey for the microneedle patch preference, 100% of participants answered “no” when asked about pain induced by the patch.

## 4 DISCUSSION

The present clinical split‐face study investigated the efficacy of microneedle patches for improving nasolabial folds. The microneedle patch with HA solution and the microneedle patch alone both significantly improved the Merz esthetics scores for nasolabial folds when evaluated by dermatologists. Notably, both groups of patients showed significant improvement in their scores, and improvements in wrinkles were observed from week 4 to the end of the study, with no significant difference in wrinkle improvement between the two groups. The low (1.8%) concentration of HA solution might be the reason for this. Theoretically, both microneedle patch and 1.8% HA solution can improve wrinkle. However, the effect of microneedle patch might be greater than 1.8% HA. Thus, there is no significant difference in wrinkle improvement between the two groups. In contrast, the satisfaction scores in the microneedle patch plus HA solution group were greater than those in the microneedle patch alone group when evaluated by the participants themselves. This improvement may have resulted from: (1) percutaneous microtrauma by the microneedle patch and (2) increased permeability of the active ingredients via the channel created by the microneedles.

Many previous studies have demonstrated the usefulness of substance delivery across the skin using microneedle devices. For example, Tammi et al. demonstrated that cutaneous microtrauma itself could stimulate the synthesis of epidermal HA via upregulated hyaluronan synthase expression.^7^ In dermatology, medical needling achieves a normalization of the skin color and an adjustment to healthy skin after repetitive treatments of hypertrophic burn scars.^8^ Kim et al. have demonstrated that microneedle patches can be used efficiently in cosmetics given their patient usability, safety, and effectiveness in wrinkle improvement.^9^ Microtrauma by tiny needles and subsequent tissue regeneration caused induction of collagen synthesis, deposition, and therefore enabled dermal rejuvenation and improved skin appearance.^10^, ^11^ Moreover, it has also been shown that the needling device itself influences vascularization by stimulating angiogenesis in the healing cascade of the postneedling wound. Specifically, the penetration of the epidermis leads to microtrauma and intradermal bleeding through the parenchymal canals without impairing the basal layers of stem cells with regenerative capacity. The induction of percutaneous collagen then reinforces endogenous potential for regeneration, and a modified wound healing cascade allows for an increased expression of growth factors, such as vascular endothelial growth factor^12^ and transforming growth factor beta (TGF‐β),^13^ both of which are important for the wound‐healing processes of angiogenesis and the differentiation of cells. Medical needling stimulates both gene expression and the proliferation of skin cells, which are essential for dermal remodeling. When percutaneous collagen is induced, the TGF‐β signal transduction pathway is altered, as TGF‐β3 reaches high levels of expression during the initial wound healing phase. This factor allows for scarless collagen synthesis and wound healing.^14^, ^15^ Further, the postneedling cascade triggers the formation of a physiological lattice‐work collagen matrix of collagen type I instead of type III, which exhibits parallel orientation and is less stable.^16^

In conclusion, the use of a microneedle patch alone has the same efficacy as a microneedle patch plus 1.8% HA solution for the improvement of wrinkles at the nasolabial folds. Notably, the microneedle patches alone hold promise for wrinkle improvement with a favorable safety profile. Since the current study focused only on Thai female participants and a low concentration of noncross‐linked HA solution, further clinical trials with a larger population, a wider range of participant skin types, and higher concentrations, or possibly a cross‐linked type of HA solution, will be beneficial to extend these results. In addition, a long‐term follow‐up study to explore the durability of the observed effects is warranted.

## CONFLICT OF INTEREST

The authors declare that there is no conflict of interest that could be perceived as prejudicing the impartiality of the research reported.

## ETHICS STATEMENT

The authors confirm that the ethical policies of the journal, as noted on the journal's author guidelines page, have been adhered to, and the appropriate ethical review committee approval has been received. The Institutional Review Board of the Human Research Ethics Committee of Thammasat University (Medicine) approved all study protocols (number: MTU‐EC‐OO‐0‐078/64, November 25, 2021) and relevant supporting data. Written informed consent was obtained from all participants.

## Data Availability

The data that support the findings of this study are available from the corresponding author upon reasonable request.
